# Chronic inflammation triggered by the NLRP3 inflammasome in myeloid cells promotes growth plate dysplasia by mesenchymal cells

**DOI:** 10.1038/s41598-017-05033-5

**Published:** 2017-07-07

**Authors:** Chun Wang, Can-Xin Xu, Yael Alippe, Chao Qu, Jianqiu Xiao, Ernestina Schipani, Roberto Civitelli, Yousef Abu-Amer, Gabriel Mbalaviele

**Affiliations:** 1 Division of Bone and Mineral Diseases, Washington University School of Medicine, St. Louis, Missouri USA; 2 Department of Pathology and Immunology, Washington University School of Medicine, St. Louis, Missouri USA; 3 University of Michigan, Ann Harbor, Michigan USA; 4 Department of Orthopaedic Surgery, Washington University School of Medicine, St. Louis, Missouri USA

## Abstract

Skeletal complications are common features of neonatal-onset multisystem inflammatory disease (NOMID), a disorder caused by *NLRP3*-activating mutations. NOMID mice in which NLRP3 is activated globally exhibit several characteristics of the human disease, including systemic inflammation and cartilage dysplasia, but the mechanisms of skeletal manifestations remain unknown. In this study, we find that activation of NLRP3 in myeloid cells, but not mesenchymal cells triggers chronic inflammation, which ultimately, causes growth plate and epiphyseal dysplasia in mice. These responses are IL-1 signaling-dependent, but independent of PARP1, which also functions downstream of NLRP3 and regulates skeletal homeostasis. Mechanistically, inflammation causes severe anemia and hypoxia in the bone environment, yet down-regulates the HIF-1α pathway in chondrocytes, thereby promoting the demise of these cells. Thus, activation of NLRP3 in hematopoietic cells initiates IL-1β-driven paracrine cascades, which promote abnormal growth plate development in NOMID mice.

## Introduction

*NLRP3*-activating mutations cause cryopyrin-associated periodic syndromes (CAPS) of which neonatal-onset multisystem inflammatory disease (NOMID) is the most severe manifestation^1, 2^. Each of these rare, but serious cryopyrinopathy phenotypes is associated with excessive interleukin (IL)-1β and IL-18 production, unprovoked recurrent episodes of fever, urticaria-like rash, arthropathy and CNS symptoms^1, 2^. In addition, skeletal anomalies are prominent features of NOMID, which include osteopenia, bone deformities, leg length discrepancy and short stature^3–6^. These defects are associated with abnormal epiphyseal calcification and outgrowths, and growth plate disorganization, but the underlying mechanisms are not understood.

Activated NLRP3 assembles a protein complex, the NLRP3 inflammasome, which processes pro-IL-1β and pro-IL-18 into biologically active, IL-1β and IL-18, respectively^7, 8^. As other members of the inflammasome family, NLRP3 is synthesized by innate immune cells, and its expression is robustly up-regulated upon NF-κB activation^9, 10^. Activating-mutations of NLRP3 causing cryopyrinopathies have been linked to abnormal NLRP3 function in myeloid cells^11, 12^. The NLRP3 inflammasome is also active in mesenchymal cells, including osteoblasts and chondrocytes^6, 13, 14^. These cells maintain skeletal integrity through the production, organization and mineralization of the extracellular matrix; but aberrant activities of these cells in pathologic conditions such as osteoarthritis can cause degenerative hypertrophy such as osteophytes and Heberdeen’s nodes. Indeed, enhanced cAMP-dependent protein kinase activity and Wnt signaling in stromal cells/osteoblasts may contribute to the tumor-like phenotype of bony outgrowths in NOMID patients^15, 16^; and hyper-activation of the NLRP3 inflammasome is presumed to promote chondrocyte apoptosis, thereby contributing to deafness in CAPS patients^6, 17^. Thus, it is possible that growth plate and epiphyseal dysplasia in NOMID may arise from aberrant NLRP3 actions in mesenchymal cells in addition to indirect effects via innate immune cells.

Consistent with the NLRP3 inflammasome’s role in over-secretion of IL-1β and IL-18 in CAPS patients^18^, IL-1 biologics are used for the treatment of these disorders^19^. Intriguingly, recent reports indicate that skeletal lesions in NOMID patients progress despite treatment with IL-1β blocking agents, while other symptoms related to systemic inflammation rapidly resolve^20–23^. These observations suggest that multiple effectors of this inflammasome influence the development of skeletal abnormalities. This view is consistent with the emerging evidence that the NLRP3 inflammasome signaling cascades promote the processing of numerous substrates, including poly(ADP-ribose) polymerase 1 (PARP1), a protein that negatively regulates bone resorption through inhibition of osteoclast differentiation^24–27^. Thus, the mechanisms leading to the skeletal abnormalities in cryopyrinopathies are still unclear.

NOMID mice in which NLRP3 is activated globally phenocopy the skeletal defects of the human disorder, but the underlying mechanisms remain unknown. In this study, we conditionally activated the NLRP3 inflammasome either in the mesenchymal or hematopoietic lineage. We found that activation of this inflammasome in myeloid cells, but not mesenchymal cells in mice causes inflammation, and growth plate and epiphyseal dysplasia through IL-1β- dependent mechanisms.

## Results

### Constitutive activation of the NLRP3 inflammasome in mesenchymal cells does not cause inflammation or abnormal growth plate development

We have previously reported a murine model of NOMID in which NLRP3 was constitutively activated globally as a result of the mating of *Nlrp3* ^*fl*(*D301N*)/+^ mice with mice expressing Cre under the control of the *Zona pelucida 3* (*ZP*) promoter (NLRP3^ZP^)^14^. D301N is the ortholog of the D303N mutation that occurs in NOMID patients. *Nlrp3* ^*ZP*^ mice exhibited several features of NOMID patients, including systemic inflammation and disorganized growth plate and epiphysis^14^ (Figure S1A). In agreement with these findings, arthropathy and osteolysis have also been reported recently in mice in which the endogenous *Nlrp3* locus was replaced by the human mutated *NLRP3* locus, also referred to as humanized mice^28^. To determine the contribution of chondrocyte to this phenotype, we generated mice in which the inflammasome was activated constitutively in chondrocytes by *Collagen II-Cre* (NLRP3^Col2^). *Nlrp3* ^*Col2*^ mice developed normally and were indistinguishable from neonatal and adult wild-type (WT) littermates based on histological assessment of the growth plate and epiphysis (Figure S1B–E) and micro-computed tomography (µCT) analysis of the subchondral bone (Figure S2A). Given that stromal cells/osteoblasts and chondrocytes are the only cells capable of forming the bony outgrowths found in NOMID patients^6, 13^, we therefore activated NLRP3 in common chondrocyte and osteoblast progenitors using *Twist-2/Dermo-1-Cre (DM1)* to generate *Nlrp3* ^*DM1*^ mice. Unexpectedly, growth plate development (data not shown), and bone mass accrual (Figure S2B) were comparable between WT and *Nlrp3* ^*DM1*^ mice.

**Figure 1:**
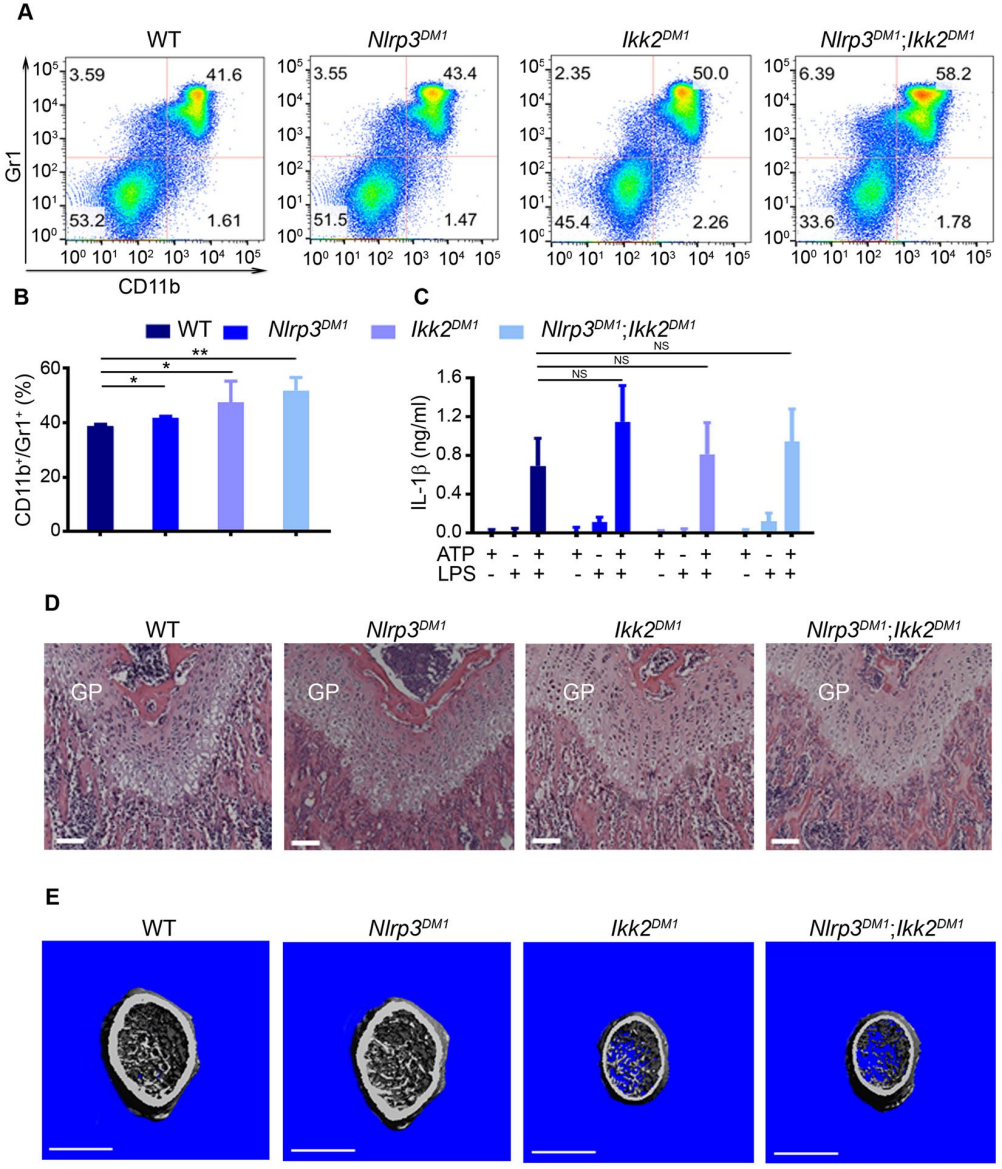
Constitutive activation of the NLRP3 inflammasome in osteochondro-progenitors does not cause inflammation and abnormal growth plate development. All data were obtained from 4-week old WT, *Nlrp3* ^*DM1*^, *Ikk2* ^*DM1*^ or *Nlrp3* ^*DM1*^; *Ikk2* ^*DM1*^ male mice (n = 3–10/genotype). (**A**) Red blood cell-depleted bone marrow cells were stained with isotype control (data not shown) or with antibodies against CD11b and Gr1. Representative flow cytometry dot plots of the CD11b^+^/Gr1^+^ myeloid cells from each genotype are shown. (**B**) The percentage of CD11b^+^/Gr1^+^ cells in bone marrow cells. Data are expressed as mean ± SEM. *P < 0.05; **P < 0.005. (**C**) IL-1β levels in conditioned media from BMM treated with 100 ng/ml LPS for 3 hours, then with 5 mM ATP for 30 minutes. Data are expressed as mean ± SD of 3 independent experiments carried out in triplicates. NS, not significant. (**D**) H&E staining. GP, growth plate. Scale bar, 100 µm. (**E**) 3D μCT reconstruction of distal femoral metaphysis. Scale bar, 1 mm.

NLRP3 expression in myeloid cells is controlled by NF-κB^9, 10^, which is activated predominantly by IKK2 in inflammatory conditions^29^. We therefore hypothesized that lack of skeletal abnormalities in *Nlrp3* mice might be related to the so-called priming signals, which are necessary to activate NF-κB; perhaps the priming signals are too low in osteochondro-progenitors in homeostatic states. We tested this premise by generating *Nlrp3* ^*fl(D301N)/*+^; *Ikk2* ^*fl/fl*^; *DM1-Cre* (*Nlrp3* ^*DM1*^; *Ikk2* ^*DM1*^) mice, in which the NF-κB pathway is also constitutively activated^30^. Despite efficient recombination of the mutant *Nlrp3* allele in epiphyseal cartilage (Figure S2C), expression of NLRP3 and IL-1β (Figure S2D), and IL-18 (data not shown) in samples from 4-week-old WT mice was not different than in *Nlrp3* ^*DM1*^, *Ikk2* ^*DM1*^ or *Nlrp3* ^*DM1*^; *Ikk2* ^*DM1*^ mice. Given that Gr1 and CD11b are highly expressed by neutrophils, and neutrophilia occurred in cryopyrinopathies^14, 25, 28, 31^, we focused on the expression of these markers to assess inflammation. The percentage of Gr1^+^/CD11b^+^ cells was increased slightly, but significantly in *Nlrp3* ^*DM1*^, *Ikk2* ^*DM1*^ or *Nlrp3* ^*DM1*^; *Ikk2* ^*DM1*^ mice relative to WT mice (Fig. 1A and B). This increase was also significant between *Nlrp3* ^*DM1*^; *Ikk2* ^*DM1*^ and *Nlrp3* ^*DM1*^ mice, but not *Nlrp3* ^*DM1*^; *Ikk2* ^*DM1*^ and *Ikk2* ^*DM1*^ mice, suggesting the dominance of IKK2 actions, some of which were unrelated to the NLRP3 inflammasome. The inability of NLRP3^DM1^ to induce adequate inflammation *in vivo* was consistent with the equivalent levels of IL-1β induced *in vitro* by TNF-α in bone marrow stromal cells, the precursors of chondrocytes and osteoblasts, isolated from WT, individual or compound mutant mice (data not shown). IL-1β production by bone marrow macrophages (BMM) *in vitro* was also not different among genotypes (Fig. 1C), as expected.

Histological assessment of the growth plate revealed no apparent patterning defects between WT and mutant mice (Fig. 1D). Likewise, subchondral bone development was also unaffected, though bone mass was lower in *Ikk2* ^*DM1*^ and *Nlrp3* ^*DM1*^; *Ikk2* ^*DM1*^ mice compared to WT or *Nlrp*3^*DM1*^ mice (Fig. 1E and Figure S2E). Collectively, these results indicate that some bone effects of IKK2 are NLRP3 inflammasome-independent. In addition, activation of this inflammasome in mesenchymal cells does not cause inflammation or skeletal abnormalities, implying that cartilage dysplasia in NOMID mice is not triggered by chondrocyte autonomous actions of the inflammasome.

### Constitutive activation of the NLRP3 inflammasome in myeloid cells causes inflammation and growth plate defects independently of PARP1

**Figure 2:**
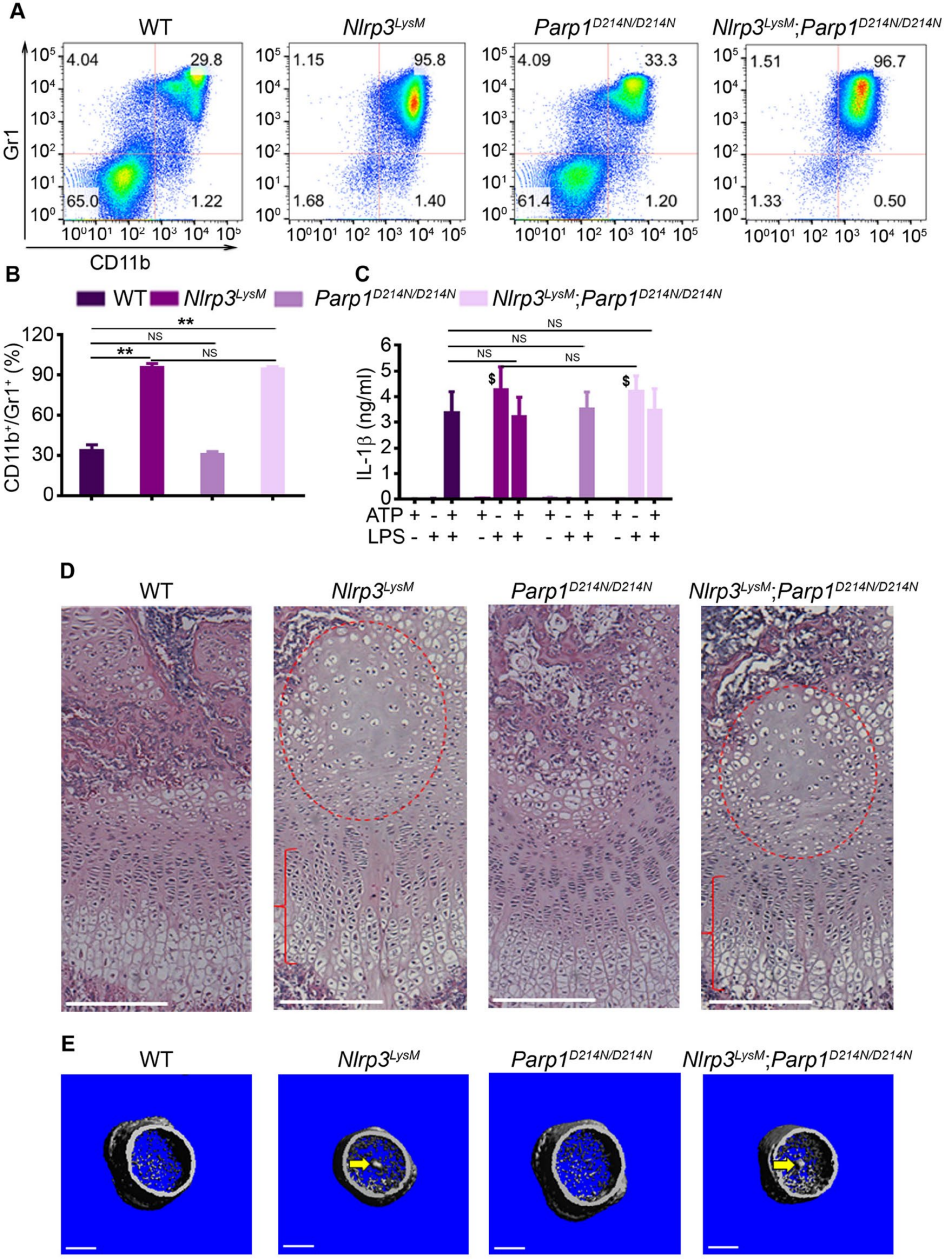
Constitutive activation of the NLRP3 inflammasome in myeloid cells causes inflammation and growth plate defects independently of PARP1. All data were obtained from 2-week old WT, *Nlrp3* ^*LysM*^, *Parp1* ^*D214N/D214N*^ or *Nlrp3* ^*LysM*^; *Parp1* ^*D214N/D214N*^ male mice (n = 3–4/genotype). (**A**) Red blood cell-depleted bone marrow cells were stained with isotype control (data not shown) or with antibodies against CD11b and Gr1. Representative flow cytometry dot plots of the CD11b^+^/Gr1^+^ myeloid cells from each genotype are shown. (**B**) The percentage of CD11b^+^/Gr1^+^ cells in bone marrow cells. Data are expressed as mean ± SEM. **P < 0.005. NS, not significant. (**C**) IL-1β levels in conditioned media from BMM treated with 100 ng/ml LPS for 3 hours, then with 5 mM ATP for 30 minutes. Data are representative of at least three independent experiments and expressed as mean ± SEM. ^$^P < 0.001 vs. WT + LPS. (**D**) H&E staining. Red circle and bracket indicate areas of hypocellularity within the enlarged center of the epiphysis and growth plate disorganization, respectively. Scale bar, 150 µm. (**E**) 3D μCT reconstruction of distal femoral metaphysis. Scale bar, 0.5 mm.

Poly(ADP-ribose) polymerase 1 (PARP1) is cleaved upon NLRP3 inflammasome activation, a response that is blunted by the D214N substitution in PARP1 (PARP1^D214N^) as we recently reported^24^. We also found that PARP1 regulates skeletal development^24, 32^, but whether PARP1 interacts with NLRP3 during this process remains unknown. Here, we used *Parp1* ^*D214N/D214N*^ mice to investigate the interplay between this protein and NLRP3. Consistent with our previous report^25^, activation of NLRP3 in myeloid cells driven by Cre under the control of the *lysozyme M* promoter (NLRP3^LysM^) resulted in a significant increase in the percentage of Gr1^+^/CD11b^+^ cells in *Nlrp3* ^*LysM*^ mice (Fig. 2A and B), which usually died 2–3 weeks after birth. PARP1^D214N^ expression had no impact on the survival of *Nlrp3* mice (data not shown) and never altered the profile of Gr1^+^/CD11b^+^ cells at baseline nor in response to inflammasome activation (Fig. 2A and B), suggesting that PARP1 is not involved in NLRP3-mediated inflammatory responses. Accordingly, IL-1β production induced *in vitro* by the inflammasome activator, ATP, was comparable between WT and *Parp1* ^*D214N/D214N*^ BMM (Fig. 2C). Moreover, PARP1^D214N^ failed to affect the secretion of this cytokine driven by NLRP3^LysM^, which did not require the presence of the nucleotide (Fig. 2C).

Strikingly, histological examinations of the femurs showed disorganized columns of chondrocytes with profoundly altered morphology in the growth plate of 2-week-old *Nlrp3* ^*LysM*^ mice (Fig. 2D, bracket and Figure S3A and B). The extracellular matrix protruded from the center of the growth plate towards the bone marrow cavity (Figure S3A and B, asterisk). These cartilage remnants were calcified to some extent since they were also detected by µCT imaging (Fig. 2E, arrow and S3C, arrow). In addition, the center of the epiphysis in mutant mice was enlarged and markedly hypocellular (Fig. 2D and Figure S3B, red circle) as in *Nlrp3* ^*ZP*^ mice, a phenotype caused by massive chondrocyte death^14^. In agreement with the observations above, the phenotype of *Nlrp3* ^*LysM*^ mice was unaffected by PARP1^D214N^ expression (Fig. 2D and E; Figure S3A–C). Thus, activation of NLRP3 causes severe inflammation and aberrant chondrocyte development independently of PARP1. Furthermore, the similarity of the phenotype of *Nlrp3* ^*LysM*^ and *Nlrp3* ^*ZP*^ mice^14^, but not *Nlrp3* ^*DM1*^ or *Nlrp3* ^*col2*^, suggests that cartilage abnormalities in NOMID mice are indirect consequences of NLRP3 inflammasome activation in myeloid cells.

### Constitutive activation of the NLRP3 inflammasome in myeloid cells causes inflammation and growth plate defects through IL-1 signaling

**Figure 3:**
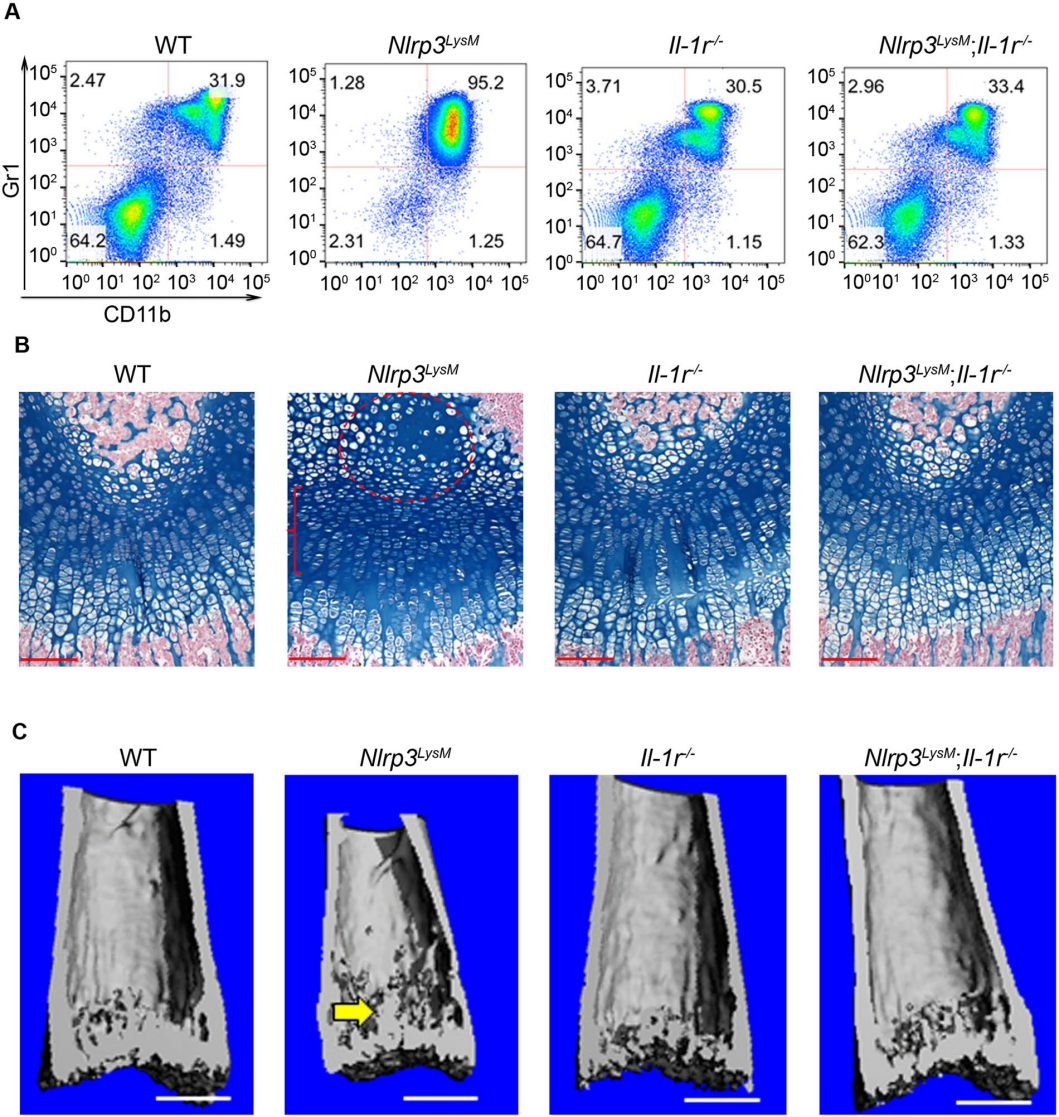
Constitutive activation of the NLRP3 inflammasome in myeloid cells causes inflammation and growth plate defects through IL-1 signaling. All data were obtained from 2-week-old WT, *Nlrp3* ^*LysM*^, *Il-1r* ^−/−^ or *Nlrp3* ^*LysM*^; *Il-1r* ^*−*/*−*^ male mice (n = 4/genotype). (**A**) Red blood cell-depleted bone marrow cells were stained with isotype control (data not shown) or with antibodies against CD11b and Gr1. Representative flow cytometry dot plots of the CD11b^+^/Gr1^+^ myeloid cells from each genotype are shown. (**B**) Safranin O staining. Red circle and bracket indicate areas of hypocellularity within the enlarged center of the epiphysis and growth plate disorganization, respectively. Scale bar, 100 µm. (**C**) 3D μCT reconstruction of distal femoral metaphysis. Scale bar, 0.5 mm.

The efficacy of IL-1 biologics in clinic for the treatment of auto-inflammatory disorders, including CAPS, provides a strong rationale for focusing on IL-1 pathway and leveraging the availability of *Il-1 receptor* (*Il-1r*) null mice to determine the role of this cytokine in NLRP3-induced skeletal manifestations. *Il-1r* ^*−*/−^ mice are unresponsive to IL-1β and develop normally. We found that the percentage of Gr1^+^/CD11b^+^ cells was comparable between WT and *Il-1r* ^*−*/−^ mice at baseline, but *Nlrp3* ^*LysM*^-induced neutrophilia was blunted by *Il-1r* ablation (Fig. 3A). Furthermore, whereas loss of *Il-1r* also had no effect on skeletal metabolism at baseline, it prevented growth plate and epiphyseal dysplasia induced by NLRP3^LysM^ (Fig. 3B and C). Thus, IL-1 signaling plays a major role in cartilage complications associated with NLRP3 hyper-activation.

### Constitutive activation of the NLRP3 inflammasome in myeloid cells causes anemia of inflammation through IL-1 signaling

**Figure 4:**
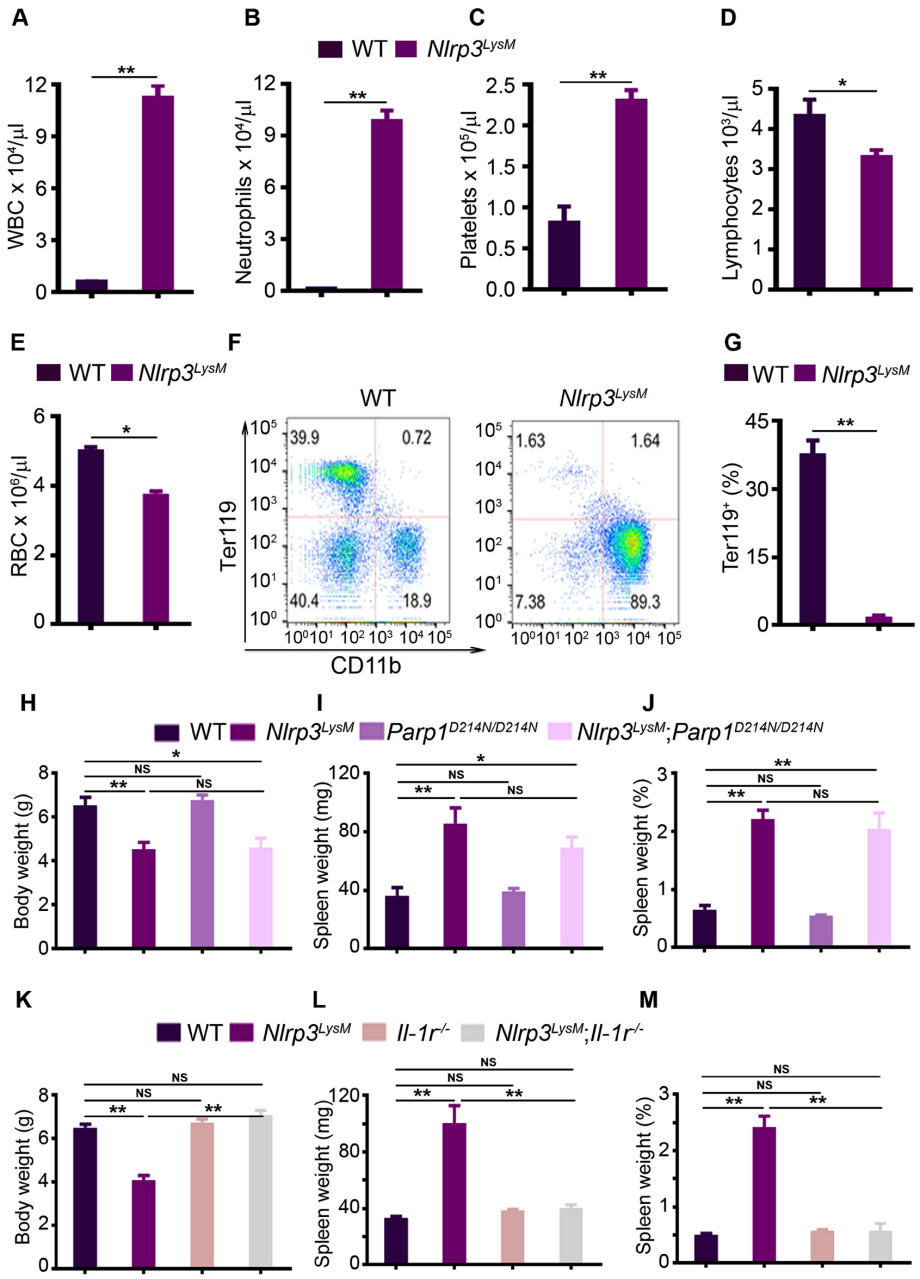
Constitutive activation of the NLRP3 inflammasome in myeloid cells causes anemia of inflammation through IL-1 signaling. All data were obtained from 2-week old WT or mutant male mice. (**A**–**E**) Cell blood counts (n = 4 mice/genotype). WBC, white blood cell counts, RBC, red blood cell counts. (**F**) Bone marrow cells (n = 4 mice/genotype) were stained with isotype control (data not shown) or with antibodies against CD11b and Ter119. Representative flow cytometry dot plots of CD11b^+^ myeloid cells or Ter119^+^ erythrocytes from each genotype are shown. (**G**) The percentage of Ter119^+^ erythrocytes in bone marrow cells. Data are expressed as mean ± SEM. *P < 0.05; **P < 0.005. (**H**) Body weight. (**I**) Spleen weight. (**J**) Percent of spleen weight (spleen weight was normalized to body weight). Quantitative data (**H**–**J**) were obtained from 5–6 mice/genotype and expressed as the mean ± SEM. *P < 0.05; **P < 0.005. (**K**) Body weight. (**L**) Spleen weight. (**M**) Percent of spleen weight. Quantitative data (**K**–**M**) were obtained from 3–5 mice/genotype and expressed as the mean ± SEM. **P < 0.005. NS, not significant.

*Nlrp3* ^*LysM*^ mice exhibited systemic inflammation characterized by an increased number of white blood cells (Fig. 4A) associated with neutrophilia (Fig. 4B), thrombocytosis (Fig. 4C), lymphopenia (Fig. 4D), and anemia (Fig. 4E). Accordingly, the bone marrow compartment of *Nlrp3* ^*LysM*^ mice contained abnormally low levels of Ter119^+^ cells (Fig. 4F and G) and CD71^+^ erythroid progenitors (data not shown). Alterations of hematopoietic cell lineages also occurred in the spleens of *Nlrp3* ^*LysM*^ mice as Gr1^+^/CD11b^+^ subsets were increased (Figure S4A and B) while Ter119^+^ cells were decreased (Figure S4C and D), responses associated with an increase in spleen size in mutant mice (Fig. 4H–M; Figure S4E and F; bottom panels). Consistent with the view that IL-1 drives the disease in *Nlrp3* ^*LysM*^ mice, anemia (Figure S4E and F; top panels) and splenomegaly (Fig. 4H–M; Figure S4E and F, bottom panels) were prevented by *Il-1r* deletion, but not PARP1^D214N^ expression. Thus, NLRP3^LysM^ expression promotes an inflammatory and anemic environment in bone marrow through the IL-1 pathway.

### Constitutive activation of the NLRP3 inflammasome in myeloid cells impairs chondrocyte responses to hypoxia

**Figure 5:**
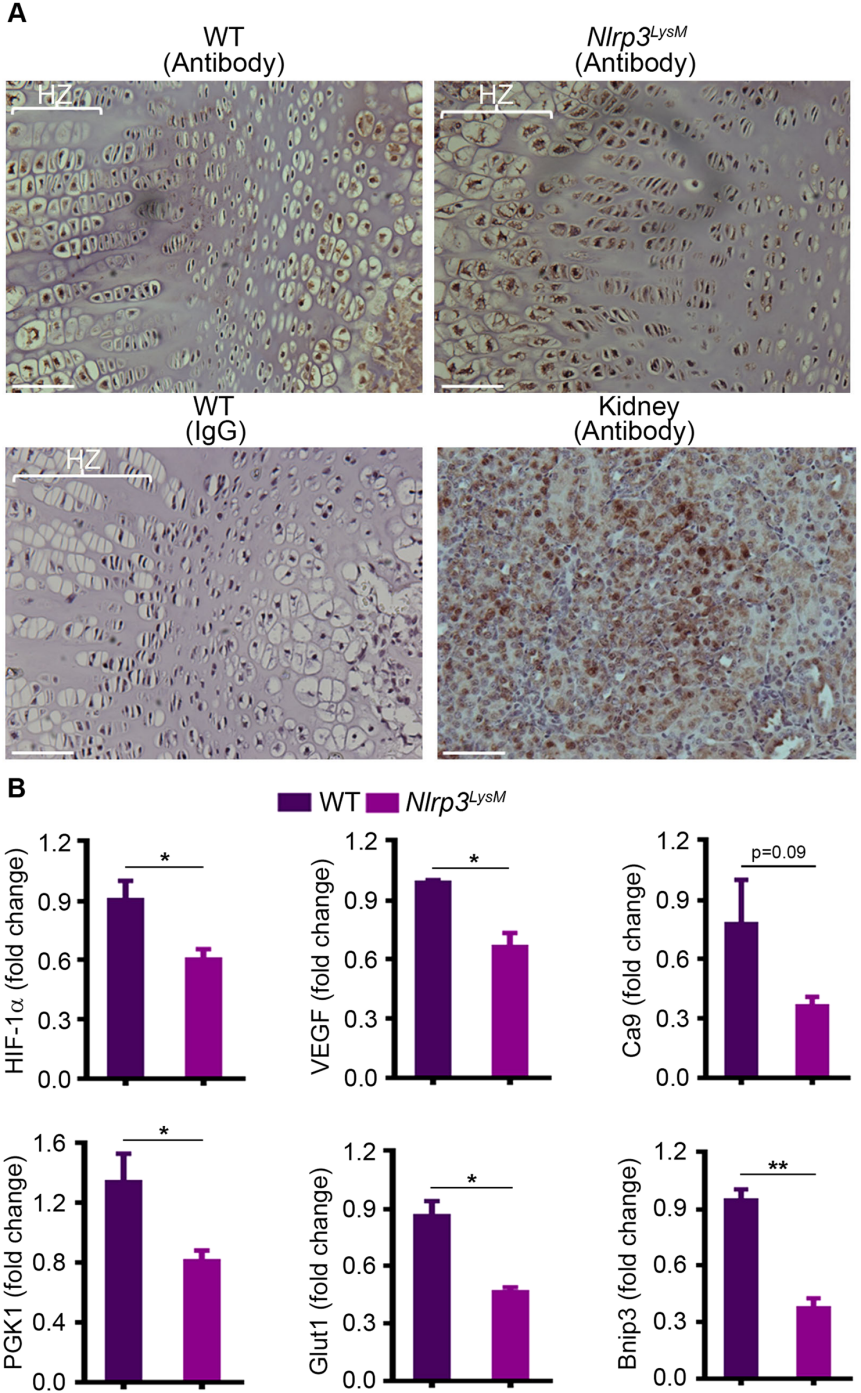
Activation of NLRP3 in myeloid cells impairs chondrocyte responses to hypoxia. (**A**) Femoral sections from 2-week-old WT or *Nlrp3* ^*LysM*^ male mice injected with hydroxyprobe were stained with IgG or hydroxyprobe antibody. A specimen from renal medulla was used as a positive control. Staining is indicated by the brown color. HZ, hypertrophic zone. (**B**) qPCR analysis of mRNA isolated from mouse epiphyses. Quantitative data were obtained from 2–3 mice/genotype and expressed as the mean ± SEM. *P < 0.05; **P < 0.005.

The center of the epiphysis of developing limbs is avascular and highly hypoxic^33^. We assessed the binding of hypoxyprobe, which detects areas of hypoxia in tissues^34^ to test the hypothesis that hypoxia may be exacerbated in the anemic environment promoted by NLRP3^LysM^ expression. We found that more chondrocytes in the epiphysis of 2-week-old *Nlrp3* ^*LysM*^ mice were labelled by the hypoxyprobe compared to sex- and age-matched WT mice (Fig. 5A, brown staining). Hence, we determined the impact of exaggerated hypoxia by analyzing the expression of hypoxia-induced factor-1α (HIF-1α) and its targets in the epiphysis. Despite hypoxia, the expression of HIF-1α and its target genes, vascular endothelial growth factor (VEGF), carbonic anhydrase 9 (Ca9), phosphoglycerate kinase 1 (PGK1), glucose transporter 1 (Glut1) and BCL2/adenovirus E1B 19 kDa protein-interacting protein 3 (Bnip3) was reduced in *Nlrp3* ^*LysM*^ mice relative to WT mice (Fig. 5B). Consistent with these results, the number of chondrocytes devoid of HIF-1α protein was higher in the epiphyses of *Nlrp3* ^*ZP*^ mice compared to WT mice (Figure S5). Thus, NLRP3 activation in myeloid cells generates paracrine signals, which inhibit the expression of HIF-1α and its targets in chondrocytes, thereby impairing responses to hypoxia.

**Figure 6:**
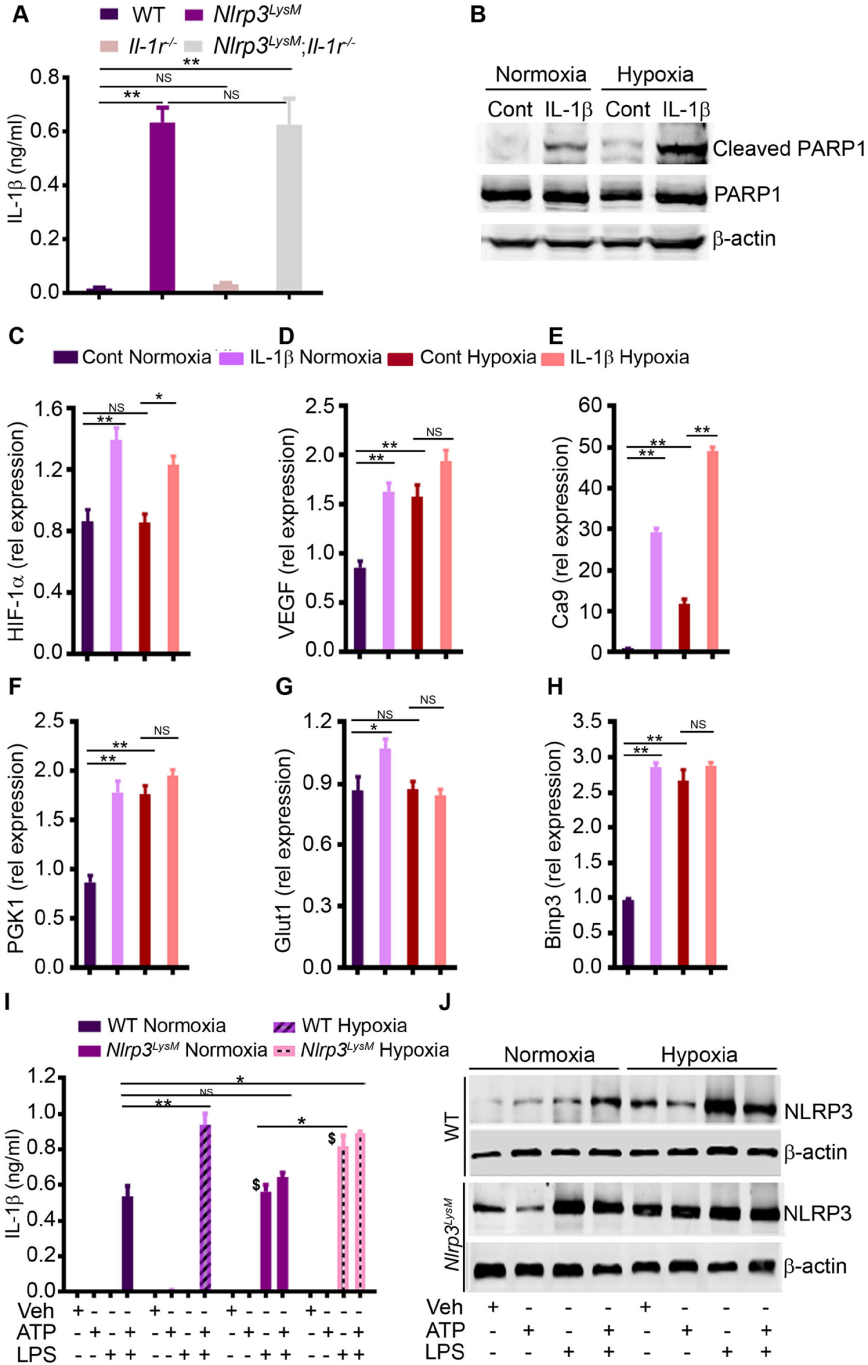
Activation of NLRP3 in myeloid cells causes excessive production of IL-1β, a cytokine that promotes chondrocyte death. (**A**) Bone marrow cells were centrifuged at 400 × g, and the supernatants were harvested for IL-1β measurement. **P < 0.005. (**B**) Western blot analysis of the effects of IL-1β on PARP1 cleavage in chondrocytes. Cells were treated with IL-1β for 24 hours. β-actin was used as loading control. Cont, control. qPCR analysis of the expression of HIF-1α (**C**), VEGF (**D**), Ca9 (**E**), PGK1 (**F**), Glut1 (**G**) and Binp3 (**H**). RNA were isolated from murine chondrocytes from the ribs of 2-day old pups. Data are expressed as the mean ± SEM. *P < 0.05; **P < 0.005; Cont, control. Rel, relative. NS, not significant. ELISA analysis of IL-1β levels in conditioned media (**I**) and Western blot analysis of NLRP3 expression (**J**) from BMM treated with 100 ng/ml LPS for 3 hours, then with 5 mM ATP for 30 minutes in normoxic or hypoxic conditions. Data are representative of 2–3 independent experiments and expressed as mean ± SEM. *P < 0.05; **P < 0.005; ^$^P < 0.001 vs. WT normoxia + LPS.

To further strengthen our proposition that IL-1β is the main culprit of NLRP3^LysM^ skeletal actions, we analyzed its expression in bone marrow. IL-1β levels were consistently higher in bone marrow supernatants from *Nlrp3* ^*LysM*^ mice relative to WT mice, but were comparable to those of *Nlrp3* ^*LysM*^; *Il-1r* ^*−/−*^ mice (Fig. 6A), suggesting that IL-1β signaling, but not production was impaired in compound mutant mice. To model the *in vivo* environment in which chondrocytes were exposed to excessive IL-1β levels, we studied the effects of IL-1β treatment on chondrocytes *in vitro*. PARP1 cleavage, an indicator of apoptosis, was induced by IL-1β, a response that was enhanced slightly when the cultures were carried out under hypoxic conditions (Fig. 6B). IL-1β also stimulated the expression of HIF-1α and its regulated genes in these cells in normoxia, but the amplitude of IL-1β-induced responses was attenuated in hypoxic conditions for most of these targets (Figure C–H). Thus, in an inflammatory and hypoxic milieu, the ability of chondrocytes to up-regulate genes of the HIF-1α pathway is compromised.

Hypoxia up-regulated IL-1β production by promoting the expression of NLRP3 and absent in melanoma 2 (AIM2)^35, 36^. Consistent with these reports, we found that hypoxia enhanced IL-1β production induced by LPS and ATP in WT and *Nlrp3* ^*LysM*^ BMM (Fig. 6I), a response that correlated with increased expression of WT and mutant NLRP3 (Fig. 6J). Likewise, IL-1β over-secretion by *Nlrp3* ^*LysM*^ BMM in the absence of ATP inversely correlated with low oxygen levels (Fig. 6I). Collectively, these data suggest that over-secretion of IL-1β by *Nlrp3* ^*LysM*^ myeloid cells, promotes chondrocyte death and causes hypoxia, which in turns sustains inflammation.

## Discussion

We find that conditional activation of the NLRP3 inflammasome in chondrocytes or osteochondro-progenitors has no detrimental impact on skeletal development in mice. Thus, the growth plate abnormalities observed in mice with global NLRP3 activation are not caused by an action of NLRP3 in the mesenchymal lineage. This observation is intriguing since NLRP3 is highly expressed in chondrocytes and osteoblasts, both of which arise from common mesenchymal progenitors. Indeed, hyper-activation of the NLRP3 inflammasome is associated with chondrocyte apoptosis, which has been hypothesized to cause deafness in NOMID patients^6, 17^. Furthermore, cultured stromal cells/osteoblasts from NOMID patients express higher levels of gene targets of the potent bone anabolic molecules, Wnts, and are more proliferative than control cells^16^. Accordingly, induced pluripotent stem cells from NOMID patients exhibit superior chondrogenic potential *in vitro* compared to normal cells^15^. Thus, hyper-active NLRP3 is apparently harmful to human, but not murine osteochondro-progenitors. Whether these dissimilar outcomes reflect differences in the experimental approaches (*in vitro* studies with human NOMID cells vs. *in vivo* studies in NOMID mice) is unclear.

We made the unexpected observation that activation of NLRP3 in myeloid cells (NLRP3^LysM^) causes not only the common symptoms of CAPS, such as excessive IL-1β production and systemic inflammation, but also cartilage anomalies. The phenotype of *Nlrp3* ^*LysM*^ mice resembles that of mice broadly expressing the transgene (NLRP3^ZP^), though it is more penetrant in *Nlrp3* ^*ZP*^ mice. More importantly, cartilage dysplasia in *Nlrp3* ^*LysM*^ and *Nlrp3* ^*ZP*^ mice recapitulates several features of the human disease, including growth plate disorganization, epiphyseal hypocellularity and perinatal onset of the lesions, which are prominent in the distal femoral epiphysis^5^. In addition, murine growth plate protrusions are abnormally calcified similar to the human counterparts as they contain components of cartilage and bone extracellular matrices, based on alcian blue staining and µCT analysis, respectively. Given that the enlargement of the epiphyses in NOMID children worsens overtime, it is tempting to speculate that these protrusions may represent the early stage of the changes seen in humans as tissue assessments in mutant mice are confined to immature skeleton before the inevitable death of these mice around 2–3 weeks of age.

All symptoms in *Nlrp3* ^*LysM*^ mice, including systemic inflammation, death and growth plate dysplasia are apparently abrogated upon *Il-1 receptor* ablation, but not stabilization of PARP1, a downstream target of inflammasome cascades and a negative regulator of bone resorption^24^. The role PARP1 in NLRP3^LysM^-mediated bone resorption is worth investigating, but is beyond the scope of this manuscript. Our results suggest that IL-1β is responsible for NLRP3-induced events investigated in this study; others find that genetic or pharmacological inhibition of IL-1 and/or IL-18 signaling provides partial efficacy in mouse disease models of MWS and FCAS^37^. These differences, which relate to the severity of residual disease, may stem from variables such as the efficiency of Cre drivers and mouse backgrounds. In fact, and most importantly, IL-1 blocking drugs are efficacious in resolving CAPS-associated inflammatory symptoms, but not bony outgrowths in human patients. In contrast, as noted above, all symptoms in *Nlrp3* ^*LysM*^ mice, including cartilage defects are prevented by inhibiting IL-1 signaling. Thus, skeletal lesions respond differently to inhibition of IL-1 signaling. However, it is likely that the absence of lesions in NOMID mice is a result of blockade of IL-1 signaling during development before the onset of growth plate dysplasia. Hence, it is plausible that early diagnosis and therapeutic intervention with IL-1 drugs in humans may prevent the development of skeletal manifestations.

The epiphysis of NOMID mice display pronounced hypoxia compared to WT littermates. Although the center of the epiphysis in developing bones is normally hypoxic^33^, our results show that hypoxia is exacerbated in mutant limbs by the excessive amounts of IL-1β, which cause severe anemia of inflammation. Accordingly, chronic exposures to IL-1β are known to cause anemia through multiple mechanisms^38^, including attenuation of proliferation and differentiation of erythroid progenitors^39^, inhibition of erythropoietin production and signaling^40^, promotion of the biosynthesis of ferritin and down regulation of the expression of ferroportin^41, 42^, which enable the storage and the release of iron, respectively, by cells such as macrophages and hepatocytes. The specific mechanisms of anemia in NOMID mice are unclear, but this anomaly is associated with inflammation, and diminished expression of HIF-1α and its target genes, events that are associated with chondrocyte death. Although the sequence of events between chondrocyte loss and impaired hypoxic responses in the *Nlrp3* ^*LysM*^ model is unsettled, our results are consistent with the severe growth plate disorganization and hypocellularity occurring in mice lacking HIF-1α. Thus, irrespective of the hierarchy of the biological events, we posit that excessive amounts of IL-1β produced by hematopoietic cells create a cytotoxic environment in bone, thereby compromising the development of osteo-chondroprogenitors.

In conclusion, we report that IL-1β over-secretion caused by activated NLRP3 inflammasome in myeloid cells, but not mesenchymal cells, drives anemia, and hypoxia in the bone environment, ultimately promoting chondrocyte death, and the development of abnormal growth plate and epiphysis. Despite some dissimilarity in the phenotype of NOMID in humans and mice, growth plate disorganization and epiphyseal hypocellularity are common features of this disorder in both species. Thus, our results showing that disease manifestations in NOMID mice are prevented by IL-1 receptor ablation have clinical implications as they suggest that therapeutic intervention with IL-1 drugs prior to the onset of skeletal lesions may prevent the development of these devastating complications in humans.

## Methods

### Mice

IL-1 receptor-deficient (*Il-1r* ^−/−^) mice were purchased from The Jackson Laboratory. *Nlrp3* ^*fl(D301N)/*+^ mice^14^, *Parp1* ^*D214N/D214N*^ mice^24^, *Ikk2* ^*fl/fl*^ mice^30^, *lysozyme M* (*LysM*)-*Cre* mice^43^, *collagen II* (*Col2*)-*Cre* mice^44^ and Dermo-1 (*DM1*)-*Cre* mice^45^ have been previously described. All mice were on the C57BL6 background, and mouse genotyping was performed by PCR. All procedures were approved by the Institutional Animal Care and Use Committee (IACUC) of Washington University School of Medicine in St. Louis. All experiments were performed in accordance with the relevant guidelines and regulations described in the IACUC-approved protocol #20160245.

### Bone mass and microstructure

Femoral bone structure was analyzed by micro-computed tomography (µCT) system (μCT 40; Scanco Medical AG, Zurich) as previously described. Briefly, femora from male mice were stabilized in 2% agarose gel, and µCT scans at 45 kVp (2-week old bones) and 55 kVp (4-week old and older bones) were taken along the length of the femurs as previously described^14, 24, 25^. The volume of interest analyzed was located just proximal to the growth plate of the femur, spanning a height of 350 µm each for the metaphyseal region.

### Histology, immunohistochemistry and *in situ* hybridization

Spleens were harvested and weighted immediately. Long bones were fixed in 10% formalin, decalcified in 14% (w/v) EDTA pH 7.2 for 10–14 days at room temperature, embedded in paraffin, sectioned at 5 μm thickness and mounted on glass slides. The sections were stained with H&E, safranin O or alcian blue as described previously^14^. For immunofluorescence, sections were incubated with 1% hyaluronidase (Sigma) for 30 minutes at 37 °C, rinsed with PBS and blocked with 10% goat serum for 1 hour at room temperature. The sections were then incubated overnight at 4 °C with rat polyclonal type II collagen antisera or a rabbit polyclonal anti-IIA antibody that recognizes the exon 2-encoded cysteine-rich domain within the NH2-propeptide of type II procollagen as previously described^14, 46^. After washes in PBS, the sections were incubated for 1 hour at room temperature with secondary antibodies conjugated to either Alexa 488 or Alexa 594 fluorescent dyes (Invitrogen). Following rinses in distilled water, DAPI containing mounting solution was applied to each tissue section. A Nikon Eclipse E800 fluorescence microscope was used to view the images. HIF-1α immunohistochemistry was carried as previously described^47^. *In situ* hybridization analyses were performed as previously described^48^ using ^35^S-labeled riboprobes on paraffin sections of bone specimens.

### Hypoxyprobe labeling

Two-week-old wild type mice were injected intraperitoneally with 60 mg/kg hypoxyprobe-1 (pimonidazole HCl, Hypoxyprobe, Inc.) in PBS. Bones and (kidneys as a positive control) were isolated 75 minutes later and fixed overnight at 4 °C before further processing as previously described^34^. Mice that did not receive hypoxyprobe were analyzed in parallel to serve as a negative control for hypoxyprobe antibody specificity. Detection of hypoxyprobe binding was performed using the Hypoxyprobe-1 Plus Kit (Hypoxyprobe, Inc.) with the fluorescein isothiocyanate (FITC)-conjugated antibody diluted 1:100 in blocking buffer and employing a peroxidase-conjugated anti-FITC secondary antibody at 1:100.

### Peripheral blood and bone marrow analyses

Complete blood counts were performed by the Washington University School of Medicine DCM Diagnostic Laboratory as previously described^25^. Bone marrow cells were flushed out as previously described^25^, and photographed.

### Cell cultures

Bone marrow macrophages (BMM) were obtained by culturing mouse bone marrow cells in culture media containing a 1:25 dilution of supernatant from the fibroblastic cell line, CMG 14–12, as a source of M-CSF^49^, a mitogenic factor for BMM, for approximately 5 days in a 10-cm dish as previously described^25^. Nonadherent cells were removed by vigorous washes with PBS, and adherent BMM were detached with trypsin-EDTA, and cultured in culture media containing a 1:50 dilution of CMG at 5–10 × 10^3^/well in a 96-well plate (for IL-1β analysis in conditioned media by ELISA) or 8 × 10^5^/well in a 6-well plate (for Western blot analysis). Chondrocytes were isolated from the ribs of 2-day old pups as previously described^50^, and plated at 1 × 10^6^/well in a 6-well plate in DMEM media containing 10% FBS.

To determine cell response in normoxic and hypoxic conditions, BMM treated with 100 ng/ml LPS for 3 hours, then with 5 mM ATP for 30 minutes or chondrocytes exposed to 10 ng/ml IL-1β (R&D Systems) for 24 hours, were maintained at 37 °C in a humidified atmosphere of 5% CO_2_, 20% O_2_ (normoxia) or 5% CO_2_, 2% O_2_ (hypoxia).

### mRNA expression analysis

Total RNA was harvested from tibia, epiphysis or cultured chondrocytes using RNeasy Plus Mini Kit (Qiagen). Complementary DNA was then synthesized with iScript reverse transcription kit (Bio-Rad) and quantified using primers listed in Supplemental Table 1. Gene expression was analyzed by SYBR Green gene expression assay (Applied Biosystems).

### Flow cytometry

Mouse bone marrow cells were prepared as described above. Splenocytes were isolated as previously described^51^. For flow cytometry analysis of the leukocytes, red blood cells were depleted with red blood cell lysis buffer (Roche). Cells (0.5–1 × 10^6^) were incubated with Fc block (anti-mouse CD16/32, BioLegend) to block nonspecific Fc binding, stained with isotype control or FITC-anti-mouse CD11b (eBioscience) and PE-anti-mouse Ly-6G/Ly-6C (Gr1) antibody (Biolegend) according to the supplier’s instructions. For flow cytometry analysis of red blood cells, whole mouse bone marrow cells or splenocytes were stained with isotype control or APC anti-mouse Ter119 (BioLegend) and BV-421 anti-mouse CD71 antibody (BD), according to the supplier’s instructions. Flow cytometry was performed using BD LSRFortessa or BD FACSCanto II Flow Cytometer system, followed by analysis with FlowJo software (Tree Star, Inc.).

### Western blot analysis

Cell extracts were prepared by lysing cells with RIPA buffer (50 mM Tris, 150 mM NaCl, 1 mM EDTA, 0.5% NaDOAc, 0.1% SDS, and 1.0%NP-40) plus phosphatase inhibitors (2 mMNaVO4, 10 mMNaF, and 1 mMPMSF) and Complete Protease Inhibitor Cocktail (Roche). Protein concentrations were determined by the Bio-Rad method, and equal amounts of proteins were subjected to SDS-PAGE gels (8–15%). Proteins were transferred onto nitrocellulose membranes and incubated with NLRP3 antibody (1:1000; Adipogen), PARP-1, cleaved PARP-1 antibody (1:1000; Cell Signaling Technologies), or β-actin antibody (Santa Cruz Biotechnology) overnight at 4 °C, followed by a 1 h incubation with secondary goat anti-mouse IRDye 800 (Rockland) or goat anti-rabbit Alexa-Fluor 680 (Molecular Probes), respectively. The results were visualized using Li-Cor Odyssey Infrared Imaging System (LI-COR Biosciences).

### Immunoassays

BMM were plated at 5 × 10^4^ cells per well on a 96-well plate and maintained for 24 h in culture media containing a 1:10 dilution of CMG. Cells were treated with 100 ng/ml LPS or PBS without changing media for 3 hours, then with 5 mM ATP for 30 minutes, and conditioned media were collected. For IL-1β levels in bone marrow, flushed bone marrow were centrifuged and the supernatants were collected as described previously^25^. IL-1β levels were quantified using the eBioscience ELISA kit.

### Statistical analysis

Statistical analysis was performed using Student’s *t*-test, one-way ANOVA or two-way ANOVA with Tukey’s multiple comparisons test in GraphPad Prism 6.

## Electronic supplementary material

Supplementary Information
