# Supplementary material for: Chronic inflammation triggered by the NLRP3 inflammasome in myeloid cells promotes growth plate dysplasia by mesenchymal cells
Source: Sci Rep. 2017 Jul 7;7:4880. doi: 10.1038/s41598-017-05033-5 (PMC5501802; doi:10.1038/s41598-017-05033-5)
Supplement: Supplementary file 1 — Supplementary Information [file 41598_2017_5033_MOESM1_ESM.pdf]

Chronic inflammation triggered by the NLRP3 inflammasome in myeloid cells promotes growth plate dysplasia by mesenchymal cells

Chun Wang[1], Can-Xin Xu[2], Yael Alippe[1], Chao Qu[1], Jianqiu Xiao[1], Ernestina Schsipani[3], Roberto Civitelli[1], Yousef Abu-Amer[4], Gabriel Mbalaviele[1*].

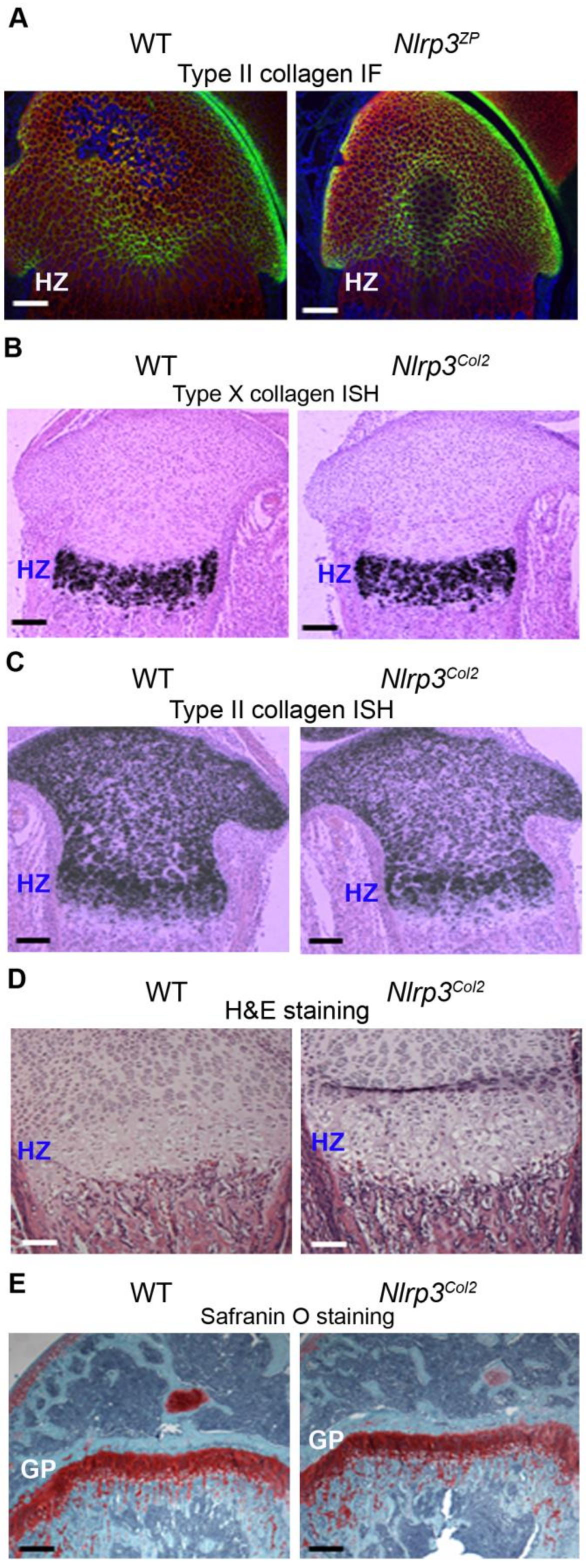

Figure S1: Constitutive activation of the NLRP3 inflammasome in chondrocytes does not cause abnormal growth plate development. (A) Immunofluorescence (IF) of femoral sections from 1-week-old mice stained for types II (green) or IIA (red) collagen, and counterstained with DAPI (blue). Scale bar, 100 µm. (B) In situ hybridization (ISH) for type X collagen was performed on sections from 3 day- old mice. Scale bar, 100 µm. (C) ISH for type II collagen was performed on sections from 3-day old mice. Scale bar, 100 µm. D) H&E staining of sections from 1-week old mice. (E) Safranin O staining of sections from 6-week old mice. Scale bar, 100 µm. HZ, hypertrophic zone; GP, growth plate.

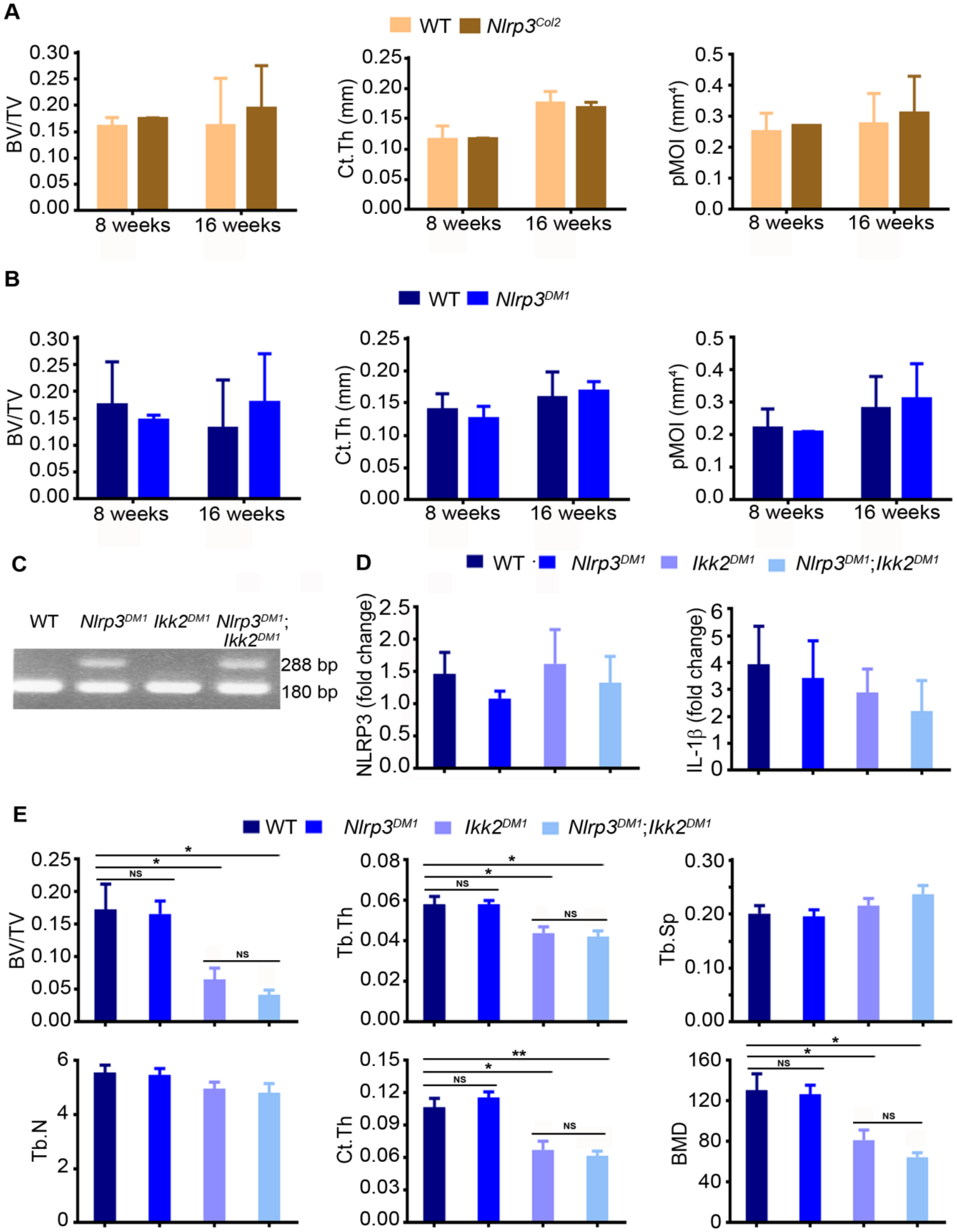

Figure S2: Constitutive activation of the NLRP3 inflammasome in mesenchymal cells does not cause bone loss. (A) µCT analysis of trabecular bone mass (BV/TV), cortical thickness (Ct.Th) and polar moment of inertia (pMOI) from 8-week and 16-week old WT or *Nlrp3*^{Col2} mice. (B) µCT analysis of BV/TV, Ct.Th or pMOI from 8-week and 16-week old WT and *Nlrp3*^{DM1} mice. (C) Analysis of allele recombination. WT, 180 bp; 288 bp, recombined *Nlrp3*. (D) qPCR analysis of mRNA from bone marrow-free tibias of 4-week WT, *Nlrp3*^{DM1}, *IKK2*^{DM1} or *Nlrp3*^{DM1};*IKK2*^{DM1} mice. (E) µCT analysis of BV/TV, trabecular thickness (Tb.Th), trabecular number (Tb.N), trabecular space (Tb.Sp), Ct.Th or bone mineral density (BMD) from the femurs of 4-week WT, *Nlrp3*^{DM1}, *IKK2*^{DM1} or *Nlrp3*^{DM1};*IKK2*^{DM1} mice. *P<0.05; **P<0.005. NS, not significant.

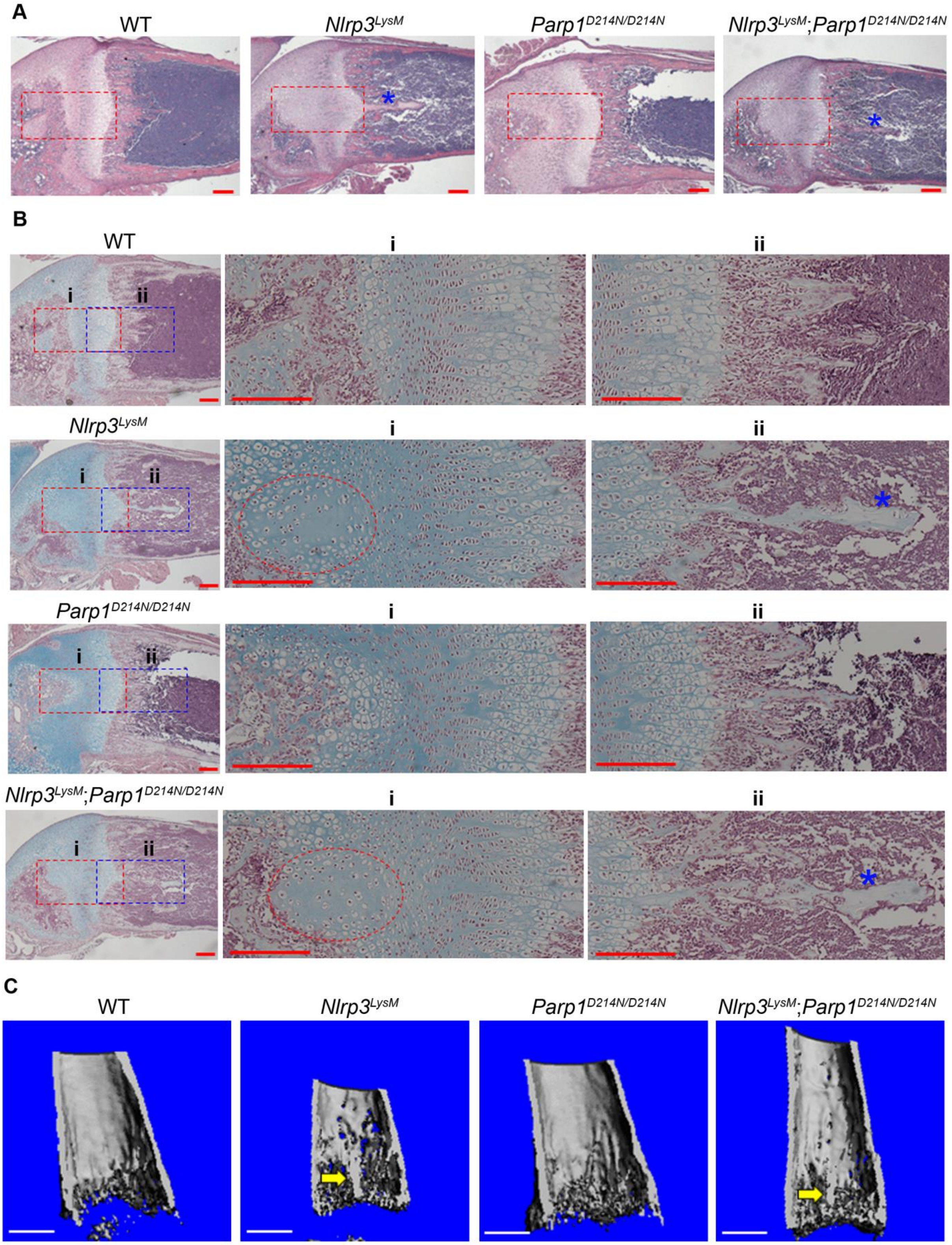

Figure S3: Constitutive activation of the NLRP3 inflammasome in myeloid cells causes growth plate defects independently of PARP1. All data were obtained from 2-week old WT, *Nlrp3* ^{LysM}, *Parp1* $^{D214N/D214N}$ or *Nlrp3* ^{LysM}; *Parp1* $^{D214N/D214N}$ male mice. (A) H&E staining of femoral sections. The red box indicates the areas that were magnified and shown in Fig. 2D. Asterisk shows growth plate protrusions. Scale bar, 150 µm. (B) Alcian blue staining of femoral sections. Higher magnifications of the boxes labelled i (red) and ii (blue) are shown in the middle and right panels, respectively. Red circle and asterisk indicate areas of hypocellularity within the enlarged center of the epiphysis and growth plate protrusions, respectively. Scale bar, 150 µm. (C) 3D µCT reconstruction of distal femoral metaphysis. The yellow arrow indicates the protrusion. Scale bar, 0.5 mm.

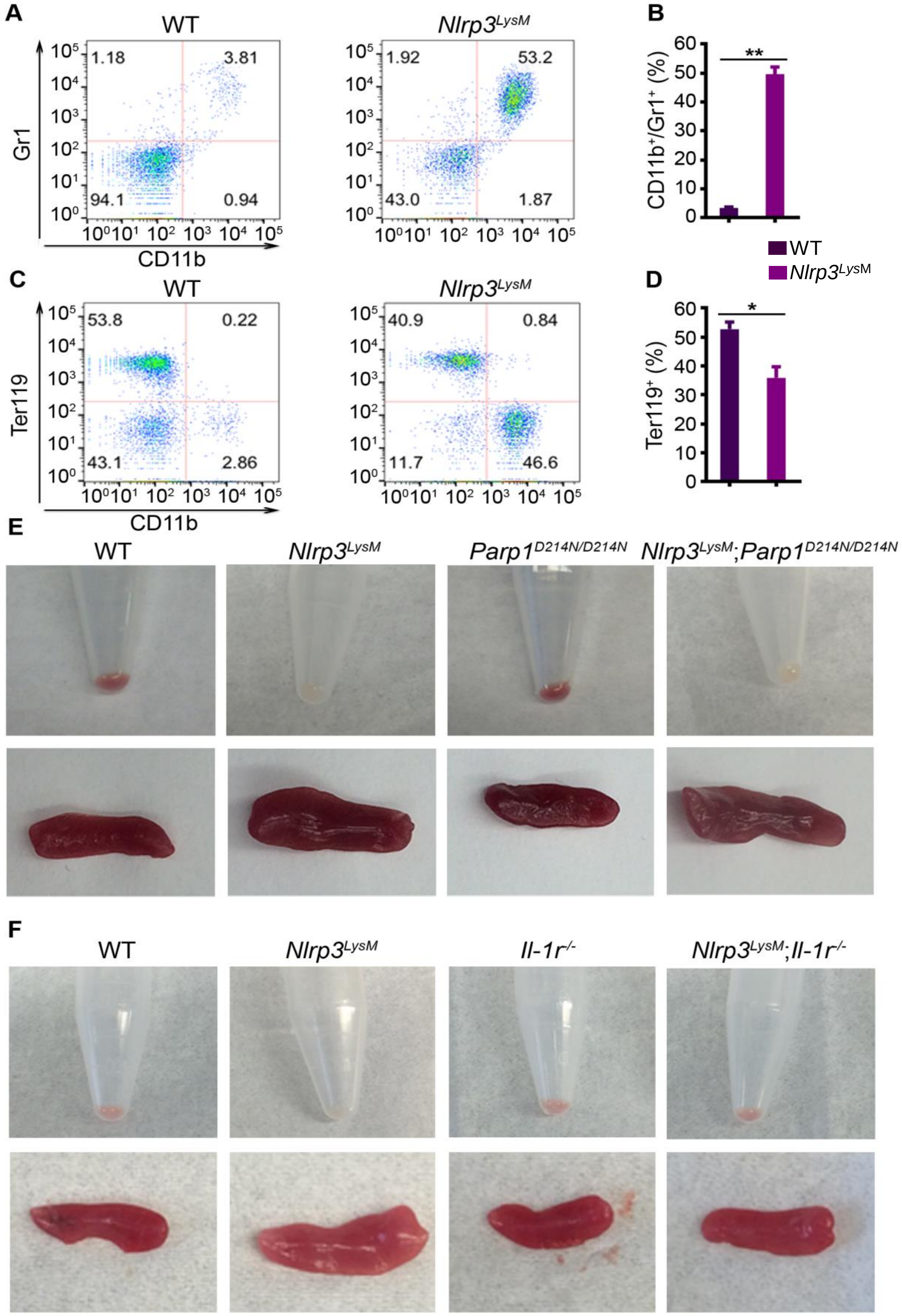

Figure S4: Constitutive activation of NLRP3 in myeloid cells causes leukocytosis, anemia and splenomegaly through IL-1, but not PARP1 pathway. All data were obtained from 2-week male mice (n=4/genotype). Splenocytes were stained with isotype control (data not shown) or with antibodies against CD11b and Gr1 (A and B), CD11b and Ter119 (C and D). (A and C) Representative flow cytometry dot plots of $CD11b^{+}/Gr1^{+}$ myeloid cells, $CD11b^{+}$ cells or $Ter119^{+}$ erythrocytes from each genotype are shown. (B and D) Quantitative data are expressed as mean ± SEM. *P<0.05; **P<0.05. (E) Pictures of bone marrow pellet (top) and spleen (bottom) from WT, *Nlrp3*^{LysM}, *Parp1*$^{D214N/D214N}$ or *Nlrp3*^{LysM}; *Parp1*$^{D214N/D214N}$ male mice. (F) Pictures of bone marrow pellet (top) and spleen (bottom) from WT, *Nlrp3*^{LysM}, *Il-1r*$^{-/-}$ or *Nlrp3*^{LysM};*Il-1r*$^{-/-}$.

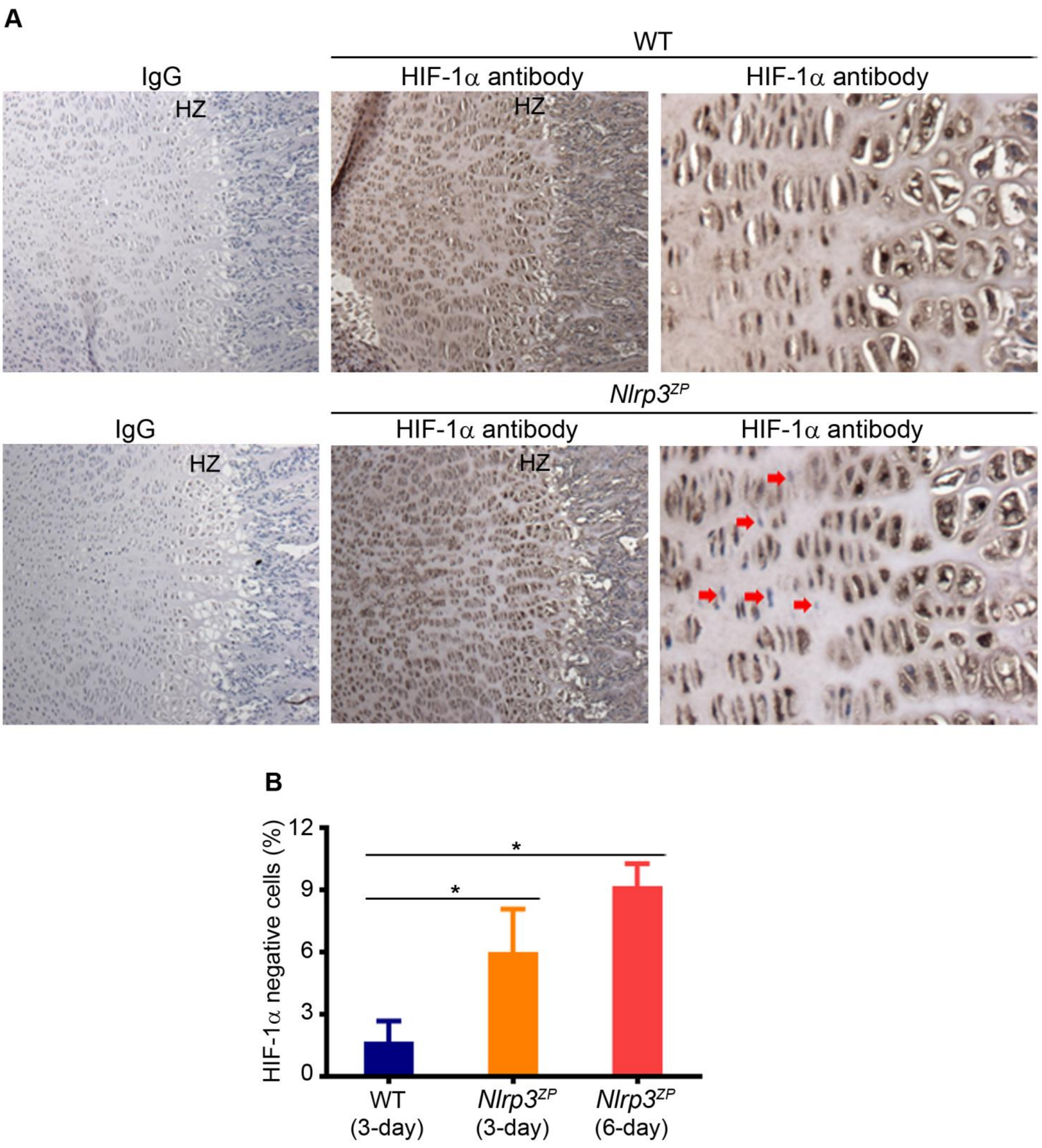

Figure S5: Constitutive activation of NLRP3 globally attenuates HIF-1α expression in the epiphysis. (A) Femoral sections from 3-day old mice WT or *Nlrp3* ^{ZP} mice were stained with isotype IgG or HIF-1α antibody. The right panels are higher views of the center of the epiphysis in the middle panels. The staining is indicated by the brown color, and the red arrows show cells with undetectable HIF-1α. HZ, hypertrophic zone. Femoral sections from 6-day old mice were also stained with isotype IgG or HIF-1α antibody (data not shown). (B) Quantitative data from 3-day old or 6-day old mice. Data are expressed as mean ± SEM. $^*P<0.05$.

Table S1. List of primers for qPCR.

Primers	Sequence
Cyclophilin B Fwd	AGCATACAGGTCCTGGCATC
Cyclophilin B Rev	TTCACCTTCCCAAAGACCAC
Nlrp3 Fwd	CCACATCTGATTGTGTTAATGGCT
Nlrp3 Rev	GGGCTTAGGTCCACACAGAA
Hif-1α Fwd	CTTGACAAGCTAGCCGGAGG
Hif-1α Rev	TCGACGTTCAGAACTCATCTTT
Vegf Fwd	GTACCTCCACCATGCCAAGT
Vegf Rev	TCGCTGGTAGACATCCATGA
Ca9 Fwd	CGTGATTCTCGGCTACAACTGA
Ca9 Rev	GGGAAGGAAGCCTCAATCGT
Pgk1 Fwd	GGAAGCGGGTCGTGATGA
Pgk1 Rev	GCCTTGATCCTTTGGTTGTTTG
Glut1 Fwd	TACACCCCAGAACCAATGGC
Glut1 Rev	CCCGTAGCTCAGATCGTCAC
Il-1β Fwd	GTGCAAGTGTCTGAAGCAGC
Il-1β Rev	CAAAGGTTTGGAAGCAGCCC
Binp3 Fwd	AAATTAAAGGGTGCGTGCGG
Binp3 Rev	AACTGCAAAGTGGGGTTCGT
